# Innovative Fermentation Approach Employing *Lachancea thermotolerans* for the Selective Production of High-Acidity Wines, Designed for Blending with Low-Acidity Counterparts to Achieve Chemically and Organoleptically Balanced Final Compositions

**DOI:** 10.3390/foods14162773

**Published:** 2025-08-09

**Authors:** Fernando Sánchez-Suárez, María del Valle Palenzuela, Antonio Rosal, Rafael Andrés Peinado

**Affiliations:** 1 Agricultural Chemistry, Soil Science and Microbiology Department, University of Córdoba, Campus of Rabanales, N-IV Road, Km 396, 14071 Córdoba, Spain; g62sasuf@uco.es; 2 Molecular Biology and Biochemical Engineering Department, University Pablo de Olavide, Utrera Road, Km 1, 41013 Seville, Spain; mvpalrui@upo.es

**Keywords:** *Lachancea thermotolerans*, acidity, lactic acid, blend, aromatic profile

## Abstract

The most notable effects of climate change on wine production are higher alcohol levels, lower acidity and changes to the regions suitable for growing grapes. One solution to acidity problems is the use of *Lachancea thermotolerans*, a yeast that produces lactic acid during fermentation, albeit at the cost of reduced aromatic complexity. A novel approach to addressing this problem is to use *L. thermotolerans* to produce wines with a very high acidity, regardless of other parameters, for subsequent blending with a control wine with a naturally low acidity. This achieves a balanced acidity while retaining the organoleptic characteristics of the control wine. This is a novel idea, as *L. thermotolerans* is not usually used in the final wine. However, the objective from the beginning is to create a blend that combines the best characteristics of the control wine with the improved acidity provided by *L. thermotolerans*. Base wines were produced by inoculating *Saccharomyces cerevisiae* 20, 40, or 60 h after inoculating *L. thermotolerans*. Base wines generally show an increase in lactic acid content and a decrease in certain key aromatic compounds, such as isoamyl acetate, 2-phenylethyl acetate, ethyl hexanoate, and ethyl octanoate. Concentrations of other compounds such as acetaldehyde and higher alcohols also increase. The base wines were then blended with a low-acidity control wine. The resulting blends exhibited higher acidity than the control wine, as well as better aromatic profiles, particularly regarding fruity and green fruit aromatic compounds, compared to base wines. Ten volatile compounds have been correlated with lactic acid production by *L. thermotolerans*, namely ethyl hexanoate; ethyl butanoate; 2-methylbutanol; ethyl heptanoate; isoamyl acetate; acetaldehyde; isobutanol; 2-phenylethanol; dodecanol; and acetoin. The first five are negatively correlated and the rest are positively correlated. Lastly, sensory analysis revealed that the blends achieved the best balance between acidity and aroma, making them the most popular with tasters.

## 1. Introduction

Climate change has a serious impact on all biological processes, with the degree of impact directly related to the expected temperature increase in each area [1]. Other factors, such as lower rainfall (although more intense), lower humidity, and changes in radiation resulting from the temperature increase, further exacerbate the stress to which the vineyard is subjected due to climate change [2]. In the context of winegrowing, this could have extremely serious consequences, as around 90% of current winegrowing regions in major winegrowing countries such as Spain, Italy and Greece could disappear by the end of the century [3].

This increase in temperature results in an earlier phenological advance in the vineyard, causing grapes to ripen during a warmer period. In other words, a 1 °C increase in the global temperature could result in a 2 °C increase in grape ripening time [4].

The main effects of climate change on grape composition include an increase in sugar content, a change in the aromatic profile towards inappropriate aromas as a result of berry shrivelling and a reduction in the acidity of musts and wines [5]. The first of these changes is mainly due to the increase in temperature, which has a certain relationship with the synthesis of sugars, although the main factor that increases their content is the evaporation of water [6]. This is due to the fact that, although the photosynthetic capacity of the vine increases with temperature and radiation, photosynthesis reaches an optimum between 25 and 30 °C, from this point it decreases as temperature increases [7]. At this point, the vine is therefore unable to synthesise sugars via photosynthesis. However, the evaporation of water from the berry increases with the rise in temperature, which favours the concentration of sugars.

Another major effect is an increase in pH and a reduction in acidity [8]. This leads to lower microbiological stability of the wine by reducing the protective effect of SO_2_ [9]. This decrease in acidity can be corrected by adding tartaric acid to the must or wine [10], but the permitted amount is limited (UE Law n° 1308/2013) [11]. Other acids, such as malic or citric, can also be added, but they are less effective at lowering pH.

A main drawback of adding tartaric acid is the subsequent precipitation of its salts in wine due to its reduced solubility in ethanol. Furthermore, excessive addition can lead to an increase in the hardness or bitterness of wines [12]. Conversely, physical methods have the disadvantage of reducing colour and tannins in wines when using cation exchange resins, as well as the high-water consumption of resins and electrodialysis for membrane regeneration [9]. However, this equipment is expensive, and small and medium-sized wineries often lack the resources to acquire it.

An alternative to chemical acidification is to use yeast strains that produce lactic or malic acid [13,14]. *L. thermotolerans*, for example, can produce lactic acid from sugars [15,16]. Several studies on *L. thermotolerans* in co- and sequential inoculations have demonstrated reductions in pH and increases in wine acidity. This contributes to wines having a higher freshness perception [16,17,18,19,20]. However, a disadvantage of using this yeast is that it increases acetic acid production. This is because the altered redox potential resulting from lactic acid production must be balanced by the production of acetic acid from acetaldehyde, which has a negative impact on the sensory quality of the wine [21]. Other negative effects include the difficulty of lactic acid bacteria carrying out malolactic fermentation due to the high lactic acid content [21], and the possible negative impact on the aromatic profile [22]. In addition, another characteristic of *L. thermotolerans* that makes co-inoculation or sequential inoculation with *S. cerevisiae* necessary is the reduced fermentative capacity, which is better than other non-*Saccharomyces* yeasts, but unable to carry out the whole fermentation, as its fermentative power reaches 10% v/v ethanol for the best strains [17].

Although there is a large body of literature on the use of *L. thermotolerans*, there have been practically no studies on using this yeast to produce wines with very high acidity for blending to moderate acidity. This study analyses the effect of using *L. thermotolerans*, followed by inoculating *S. cerevisiae* at different stages of fermentation, on wine composition, with the aim of blending to adjust acidity with the control wine.

## 2. Materials and Methods

### 2.1. Fermentation Conditions

The tempranillo grape variety from the cellar Viñas de Alange, S.A. (Alange, Badajoz, Spain) was used in this study. After harvesting the grape, they were destemmed and crushed. The must contained 235 g/L of reducing sugars, a pH of 3.7 and the titratable acidity, expressed as g/L of tartaric acid, was 5.02. Must and grape pomace were introduced in stainless stell tanks of 20 litres of capacity. Two yeasts strains were used, namely *L. thermotolerans* (Level 2 Laktia^®^) and *S. cerevisiae* (Velluto^®^), both from Lallemand Bio SL, Madrid, Spain. The inoculations were carried out as follows: (1) pure culture of *S. cerevisiae* (control); (2) *L. thermotolerans* followed by inoculation of *S. cerevisiae* after 20 h, BW20; (c) *L. thermotolerans* followed by inoculation of *S. cerevisiae* after 40 h, BW40; (d) *L. thermotolerans* followed by inoculation of *S. cerevisiae* after 60 h, BW60. The dose of yeast was 20 g/hl in all cases.

Fermentations were carried out by triplicate at 21 ± 1 °C with periodic punch-downs to keep the cap moist and facilitate the extraction of compounds from the grape skins. Fermentation progress was monitored by measuring density every 24 h until it dropped below 995 g/L. Once fermentation was complete, the grape pomace was pressed, and the obtained wine was mixed with the free-run wine. Finally, the wines were inoculated with the bacteria *Oenococcus oeni* (Lalvin VP41^®^, Lallemand) at a dose of 1 g/hL for the malolactic fermentation.

### 2.2. Coupages

Blends were created by combining the control wine with different wines in which *L. thermotolerans* had been introduced, with the aim of achieving a final wine with a titratable acidity of around 5.5 g/L. The following blends were obtained: (a) CW20: 80% control wine and 20% BW20 wine; (b) CW40: 55% control wine and 45% BW40 wine; (c) CW60: 50% control wine and 50% BW60 wine.

### 2.3. Enological Parameters

BENGEL^®^ bentonite and PROVEGET 100^®^ vegetal protein, both supplied by Agrovin S.A., were used to clarify the wines at respective doses of 25 g/L and 15 g/L. General parameters (pH, titratable acidity, volatile acidity, ethanol content and colour index) were analysed according to official procedures [23]. Malic and lactic acid content was determined using reflectometry with Reflectoquant™ equipment (Merck^®^, Darmstadt, Germany).

### 2.4. Analysis of Volatile Compounds

The volatile compounds present in the must and wine can be divided into two groups according to their concentration: major volatile compounds (≥10 mg/L) and minor volatile compounds (<10 mg/L). To carry out the analysis, three biological replicates were performed.

#### 2.4.1. Majority Volatile Compounds

Quantification of the volatile majority compounds and polyols was performed using an Agilent Technologies HP 6890 Series II gas chromatograph (Palo Alto, CA, USA), equipped with the CP-WAX 57 CB capillary column (50 m long, 0.25 mm internal diameter and 0.4 µm thick) and a flame ionisation detector (FID), following the protocol described by Peinado et al. [24].

For the analysis, 0.5 µL aliquots of wine samples (10 mL) previously prepared with 1 mL of 4-methyl-2-pentanol as internal standards (1024 mg/L) were injected. Prior to injection, tartaric acid was removed by precipitation with 0.2 g calcium carbonate and subsequently centrifuged at 300 g.

The chromatographic conditions set were as follows: a 30:1 split ratio, FID and a temperature programme starting at 50 °C for 15 min, followed by an increase of 4 °C/min until 190 °C was reached, where it was maintained for 35 min. The injector and detector temperature were 270 and 300 °C, respectively. The carrier gas used was helium, with an initial flow rate of 0.7 mL/min for 16 min, followed by an increase of 0.2 mL/min to reach a final flow rate of 1.1 mL/min, maintained for 52 min. Identification and quantification of the analysed compounds was performed by injection of standards under the same conditions as the samples. Additional information on the linear retention index (LRI) used for the identification of volatile compounds can be found in Table S2.

#### 2.4.2. Minority Volatile Compounds

The analysis of these compounds was carried out in two stages, both previously described in detail by Lopez de Lerma et al. [25].

In the first stage, an extraction procedure was carried out based on the use of stir bars, TWISTTER, with a PDMS thickness of 0.5 mm and a length of 10 mm (Gerstel GmbH, Mülheim an der Ruhr, Germany). Stir bars were placed in a vial containing 10 mL of a 1:10 diluted sample and 0.1 mL of ethyl nonanoate (0.4464 mg/L) as the internal standard. After shaking for 100 min at 1500 rpm, the stir bars were removed and placed in a desorption tube for subsequent chromatographic analysis. In the second phase, the volatile compounds were analysed by gas chromatography coupled to mass spectrometry (GC-MS), using a Gerstel TDS 2 thermal desorption system. The stirring bars, contained in the desorption tubes, were subjected to a temperature of 280 °C to release the volatile compounds in a CIS 4 PTV cooling system, programmed at 25 °C and containing a Tenax adsorption tube. Subsequently, the CIS was heated to transfer the volatile compounds to the GC-MS, which used an Agilent-19091S capillary column (30 m × 0.25 mm internal diameter and 0.25 µm film thickness). The mass detector operated in scanning mode at 1850 V, analysing a mass range between 39 and 300 amu.

For the identification of volatile compounds, analytical standards were injected under the same chromatographic conditions as the samples, comparing retention times with the Wiley spectral library. Quantification was carried out using calibration curves. Additional information on the linear retention index (LRI) for the identification of volatile compounds is available in Table S1.

#### 2.4.3. Calculation of Aromatic Series

The Odour Activity Values (OAVs) of the volatile compounds were calculated by dividing their concentrations by their corresponding odour perception thresholds.

Aromatic series are defined as groups of volatile compounds that share similar odour descriptors, and the overall value of each series is obtained by summing the OAVs of its constituent compounds. Nine aromatic series were identified: chemical, green, citrus, creamy, floral, fruity, green fruit, waxy and honey. It is worth noting that a single volatile compound may be associated with one or more aromatic series depending on its specific sensory attributes (see Table S3). Several authors have adopted this approach to reduce the number of variables and allow for simpler, more intuitive interpretation of the results [25,26,27,28,29,30].

### 2.5. Organoleptic Characterization

A panel of winery experts, comprising three women and five men, carried out an organoleptic characterisation of the wines obtained, following AENOR guidelines. Four parameters were established for evaluation: three of these (sight, taste and aroma) were evaluated on a hedonic scale from 1 to 10, where 10 is highly desirable and 1 is highly undesirable. The fourth parameter evaluated was acidity, which was assessed on a scale of −5 to 5, where 0 indicates perfect acidity, and −5 and 5 indicate very low and excessive acidity, respectively.

Prior to analysis, all samples were stored for 24 h at 4 °C. The wine samples (30 mL) were presented to the tasters at room temperature (20 °C) in standardised wine glasses (NF V09-110 AFNOR, 1995), in accordance with ISO 3591 norms [31]. The wines were poured in a random order and labelled with numerical codes. There was a one-minute break between samples.

### 2.6. Statistical Analysis

An ANOVA statistical analysis was carried out for each group of trials (base wines and coupages) separately. When significant differences were found, a post hoc Tukey analysis (*p* < 0.05) was performed to identify homogeneous groups. Cluster analysis and principal component analysis (PCA) were also performed to determine the similarity between the obtained wines based on the main determined parameters. All these analyses were performed using IBM SPSS Statistics 25 software (Armonk, New York, NY, USA).

Additionally, a heatmap with correlation coefficients was created using the open-source Python 3.9.7. programming language in the Anaconda Jupyter Project environment (Anaconda Inc., Austin, TX, USA), in an attempt to correlate the lactic acid content of the wines with the influence of *L. thermotolerans* on the profile of aromatic minority compounds.

## 3. Results and Discussion

### 3.1. Base Wines

#### 3.1.1. Enological Parameters

The values of the enological general parameters are shown in Table 1. It should be noted that the reduction in pH is independent of how long the *L. thermotolerans* yeast remains in the must, reducing it by around 0.4 points. This is due to the wines’ buffering capacity, which offsets the reduction in pH even when the acidity increases [10]. This phenomenon has also been observed in other studies with pH reductions ranging from 0.15 to 0.27 units [19,20].

Regarding lactic acid content, it increases with the residence time of *L. thermotolerans* in isolation. However, no significant differences were observed between 40 and 60 h. The literature on this topic is very disparate: some authors like Su et al. [32] have described an increase in lactic acid content for commercial strains, while for indigenous strains, an increase was observed as the time in solitary yeast increased. Other authors have reported similar final lactic acid values in wines produced using only *L. thermotolerans*, as observed in the present study [16]. In short, as several studies have shown, the production of lactic acid by yeast is conditioned by temperature, must sugar content, pH, sulphur and nitrogen availability [16,22,33,34].

Titratable acidity values are clearly influenced by lactic acid and volatile acidity values. The latter parameter increases as the time taken for sequential inoculation increases, although it remains far from the maximum limit. It is likely that one of the pathways used for synthesising lactic acid is similar to that described for the lactic fermentation of hexoses by bacteria. In this case, formation takes place from pyruvic acid and reducing power is consumed in the form of NADH during this transformation [35]. The absence of transformation of this pyruvic acid into ethanol, the normal reoxidation pathway for coenzymes, implies that wines produced with the intervention of *L. thermotolerans* have a lower ethanol content. This is analogous to glycerol–pyruvic fermentation. The redox balance must be restored, which can be achieved through the production of acetic acid, succinic acid, and glycerol. Depending on the strain, one pathway will be favoured over another [36]. In this case, higher levels of volatile acidity and glycerol were observed in the wines produced using *L. thermotolerans* (Table 1). Diethyl succinate values also increased in these wines (Table 2). Conversely, a reduction in ethanol content of up to 1% was observed in the case of inoculation with *S. cerevisiae* after 60 h. Morata et al. and Vicente et al. [19,20] found reductions of 0.45% and 0.42%, respectively, with sequential inoculations of *S. cerevisiae* over 6 and 5 days, respectively.

The malic acid values are higher in wines where sequential inoculation was carried out at 40 and 60 h. This is probably because, despite being inoculated with an *O. oeni* strain for malolactic fermentation, the process was slowed down due to the high initial lactic acid content. This phenomenon has also been reported by other researchers [21].

The increase in red colorations takes place when the pH decreases [35]. The increase in color intensity and tonality and total anthocyanins related to the influence of *L. thermotolerans* varies from 6% to 12%. Other authors [17] have described similar percentages. The reason for this increase is that a higher acidity facilitates the extraction of phenolic compounds and anthocyanins from the grape skin during the fermentative maceration phase [37].

#### 3.1.2. Volatile Compounds

Table 2 shows the volatile compounds organised by their chemical families. Noteworthy differences were observed among the major higher alcohols, particularly between 3-methylbutanol and 2-phenylethanol. The latter compound is synthesised in greater quantities by *L. thermotolerans*, especially when sequential inoculation is carried out, compared to inoculation of *S. cerevisiae* alone [36]. Among the minor alcohols, hexanol stands out, but it is synthesised during the pre-fermentative stage and is far from its threshold of perception (8 mg/L) [12]. Octanol, decanol and dodecanol are synthesised from their respective fatty acids by reduction [38]. The different behaviour observed in the wines is probably due to redox potential equilibria. It is also notable that octanol and dodecanol increases with the residence time of *L. thermotolerans* in isolation.

A distinction must be made between majority and minority esters among the esters. Ethyl lactate is a notable example of a majority ester in wines produced with *L. thermotolerans*, resulting from the esterification of the abundant lactic acid present in these wines and ethanol [10]. Minority esters are present in lower concentrations in wines fermented with *L. thermotolerans*, primarily due to the presence of ethyl esters of butanoic, hexanoic and octanoic acids, as well as isoamyl acetate. Vicente et al. [35] observed lower production of these esters in 12 *L. thermotolerans* strains compared to one *S. cerevisiae* strain, attributing this to lower fatty acid production. However, the possibility of lower esterase activity should not be ignored [39].

Aldehydes behave similarly to esters; the most abundant of these is acetaldehyde in wines produced with the help of *L. thermotolerans*. The same is true of most ketones, with acetoin levels increasing in wines fermented with *L. thermotolerans*. These increases may be due to regulation of the yeast’s redox potential, since lactic acid production generates NAD+, which accumulates in the medium and must be reduced to NADH for the process to continue [13]. This may explain why glyceropyruvic fermentation is favoured in this yeast for carrying out this reduction. The main compounds that accumulate in wine as a result of glyceropyruvic fermentation are glycerol, acetaldehyde and acetoin [13].

Finally, a slight decrease in the levels of terpenes and norisoprenoids was observed in wines fermented by *L. thermotolerans*, mainly due to higher levels of limonene and β-citronellol in control wines. This may be due to lower β-glucosidase activity, as the level of this enzyme in *L. thermotolerans* yeast appears to vary greatly depending on the strain used, with no clear trend observed within the species [39].

#### 3.1.3. Aromatic Series

As outlined in the Materials and Methods section, the volatile aroma compounds were classified into aromatic series. This approach has been adopted by several authors as a means of reducing the number of variables and allowing for simpler, more intuitive interpretation of the results [25,26,27,28,29,30]. Nine aromatic series were identified: fruity; green fruit; green; creamy; citrus; chemical; honey; waxy; and floral.

As shown in Table 3, the low values for the fruity, green fruit and waxy series of wines fermented with *L. thermotolerans* stand out, primarily due to their lower ester content. The compounds that contribute most to this decrease are ethyl octanoate, hexanoate, butanoate and isoamyl acetate. Similar results have been found by other authors [36]. In particular, one author found reductions of 44% and 36% in the content of butanoate and ethyl hexanoate, respectively, in a study involving 13 strains of *L. thermotolerans* compared to *S. cerevisiae*, irrespective of the strain used.

However, the creamy series, which relies primarily on lactones, acetoin and ethyl lactate, exhibits higher values in wines fermented by *L. thermotolerans*. The values of the floral series are also noteworthy, as they are mainly influenced by 2-phenylethanol. *L. thermotolerans* synthesises this compound in higher amounts when inoculated in sequential fermentations with *S. cerevisiae*, in response to stress caused by competition for both yeasts’ resources [36].

In short, the sum of the odour values for all the series in the same trial shows that using *L. thermotolerans* results in wines with a lower total odour activity value. This value decreases the longer the inoculation with *S. cerevisiae* is delayed, i.e., BW60 wines have a lower value than BW40 and BW40 less than BW20. However, the increase in acidity values for these wines is significantly higher. For this reason, blends were created to compensate for the lower content of volatile compounds while correcting the acidity.

### 3.2. Coupages

To improve the aromatic composition of wines produced using *L. thermotolerans* and increase their acidity compared to the control wine, several blends were created as described in the corresponding section of the Materials and Methods. To this end, blends of different proportions of base wine and control wine were made to achieve a final acidity of around 5.5 g/L. In this sense, it must be considered that the perception of acidity in wine is influenced by both pH and titratable acidity, but these two parameters affect the sensory experience in different ways. On one hand, pH reflects the concentration of free hydrogen ions and is more directly correlated with the intensity of perceived sourness. Lower pH values result in a sharper, more vivid perception of acidity. On the other hand, titratable acidity measures the amount of non-dissociated acids in the wine. It contributes more to the overall freshness and structure but is less influential than pH in immediate taste perception [40,41,42]. Furthermore, it should be noted that the structure provided by acidity is more important for perceived quality than the intensity of the acidity itself [43].

#### 3.2.1. Enological Parameters

Among the enological general parameters, significant differences are found mainly in pH, with a lower value in the blend containing a higher proportion of wine made with *L. thermotolerans* (CW20), especially in comparison with the control. The titratable acidity and lactic acid content are lower in the control wine (Table 4).

#### 3.2.2. Volatile Compounds

A comparative analysis of the concentrations of volatile compounds in the control wine and the CW20, CW40 and CW60 blends reveals distinct aroma profiles influenced by the proportion of wine fermented with *L. thermotolerans* in each blend (see Supplementary Table S1). The CW20 blend differs most from the control wine, showing significant reductions in fruit esters such as isoamyl acetate, ethyl hexanoate and ethyl octanoate. There is also a marked increase in acetaldehyde and ethyl lactate, contributing to a more oxidised and lactic profile and thus lower aromatic similarity to the reference wine.

The CW40 blend shows partial recovery of key volatile compounds such as the floral 2-phenylethanol and phenethyl benzoate. Although some esters remain below control levels, the aromatic profile is more balanced than that of the CW20 blend, approaching the sensory profile of the original wine. Finally, CW60, which contains the highest proportion of control wine, has the most balanced volatile composition. The concentrations of the main esters (such as isoamyl acetate and ethyl hexanoate) are closer to those of the control wine, and the distribution of aldehydes, esters, and floral compounds is more aligned with the control wine. In conclusion, CW60 most closely resembles the control wine in terms of aromatic composition. It achieves improved retention of fruit and floral compounds, and the improved acidity results in a balanced blend that combines freshness with a similar volatile profile to the original wine.

#### 3.2.3. Aromatic Series

Blends generally increase the aromatic values compared to the base wines, although the control still shows higher values. There were not many significant differences among the wines obtained after blending, since the longer *L. thermotolerans* remained in the base wine, the less aromatic it was. However, there was also a lower proportion of it in the respective blend (Table 5).

The values of the floral series stand out, showing higher values in the CW60 and CW40 wines than in the control wine. The most important compounds in the floral aromatic series are 2-phenylethanol, B-Damascenone, B-citronellol and 2-phenylethanol acetate. Additionally, the fruity and green fruit series show a notable increase compared to base wines made with *L. thermotolerans*. The most important compounds in these series are the ethyl esters of hexanoic, octanoic, butanoic and isobutanoic acids.

In short, the use of coupages has significantly increased the odour intensity of the wines, particularly in the ethyl ester-dependent series, such as the fruity, green fruit and waxy series. There have been average increases of 59%, 186% and 278% in the CW20, CW40 and CW60 wines, respectively, compared to their base wines, BW20, BW40 and BW60. This demonstrates the effectiveness of coupages in balancing the acidity of wines without abruptly decreasing aromatic capacity.

### 3.3. Cluster and Principal Component Analysis

Cluster analysis is a multivariate statistical technique that involves grouping subjects according to their degree of similarity. As it is a descriptive method rather than an explanatory one, it is up to the analyst to interpret the meaning of each grouping. The distance at which the different groups merge is a unitless measure indicating how similar they are to each other. Several methods can be used to carry out cluster analysis; in this study, Ward’s method was employed as it is the most effective for discriminating and defining levels of clustering [44]. In this study, general parameters related to the acidity, colour and aromatic profile (aromatic series) of the wines were used as variables.

**Figure 1:**
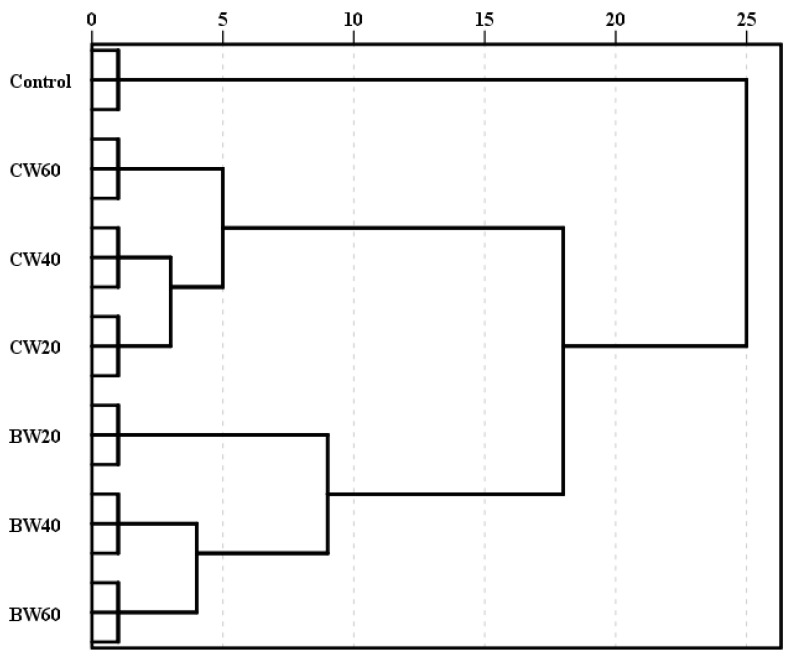
Dendrogram of cluster analysis. Control: control wine; BW20: 20 h base wine, inoculation of *S. cerevisiae* 20 h after *L. thermotolerans*; BW40: 40 h base wine, inoculation of *S. cerevisiae* 40 h after *L. thermotolerans*; BW60: 60 h base wine, inoculation of *S.* cerevisiae 60 h after *L. thermotoleran*s CW20: coupage wine made with BW20 and control wine (80–20%); CW40: coupage wine made with BW40 and control wine (55–45%); CW60: coupage wine made with BW60 and control wine (50–50%).

As can be seen in Figure 1, there are two distinct groups. The first group comprises wines in which *L. thermotolerans* participated alone for different periods of time. Within this group, the wines in which *L. thermotolerans* participated for 20 h are differentiated from the rest. The second group comprises the blends, clearly differentiating the control from the rest wines in another group. The remaining wines are grouped together, with the CW20 and CW40 wines forming one cluster and the CW60 wines forming another. Comparing the separation of the three coupage wines with that of the three base wines suggests that the former are more similar to each other.

As this type of analysis does not provide information on the variables with the greatest influence on the observed differences, a principal component analysis (PCA) was performed. This statistical method reduces the dimensionality of a dataset by converting the original variables into new, uncorrelated ones. Ideally, a small number of components should explain most of the variability in the data. The analyst must then associate the chosen components with one of the sources of variation. In this case, the classifying variables were the same as in the cluster analysis.

**Figure 2:**
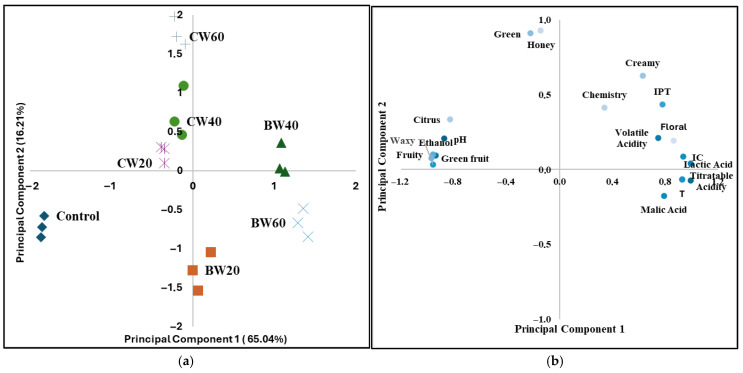
(**a**) Principal component analysis of the control, base and coupage wines; (**b**) PCA component value chart. Control: control wine; BW20: 20 h base wine, inoculation of *S. cerevisiae* 20 h after *L. thermotolerans*; BW40: 40 h base wine, inoculation of *S. cerevisiae* 40 h after *L. thermotolerans*; BW60: 60 h base wine, inoculation of *S.* cerevisiae 60 h after *L. thermotoleran*s CW20: coupage wine made with BW20 and control wine (80–20%); CW40: coupage wine made with BW40 and control wine (55–45%); CW60: coupage wine made with BW60 and control wine (50–50%).

Of the set of components obtained, the first two together explain 81.23% of the observed variability (Figure 2a). The first of these (65.02%) clearly differentiates the control wine, which is located on the negative side of the component, from the rest. The blended wines are situated around zero with the base wine BW20, while the BW40 and BW60 base wines are situated at positive values. The most important parameters in forming this component were titratable acidity, lactic acid, fruity and green fruit aromatic series (Figure 2b). The second component (16.21%) contributes to differentiate the blend wines from the base wines. The most important parameters in the formation of this component were the green and honey aromatic series.

As can be seen in Figure 2a,b, the coupage wines, located in the fourth quadrant, stand out for their green, citrus and honey aromas. Base wines fermented with *L. thermotolerans*, located in the first and second quadrants, stand out for their titratable acidity, lactic acid content, tonality and chemistry and floral series, as well as volatile acidity and colour index. Finally, the control wine, located in the third quadrant, stands out due to its higher ethanol content, as well as its higher pH values and its fruity and green fruit aromas.

### 3.4. Analysis of the Correlation Between Aromatic Compounds and Lactic Acid Production

**Figure 3:**
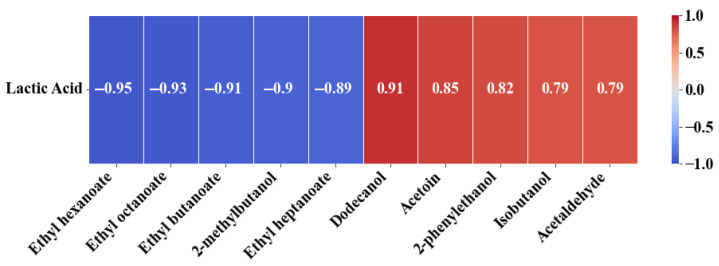
Pearson correlation values of the minor volatile compounds better related with lactic acid production.

To identify the aromatic compounds most affected by lactic acid production by *L. thermotolerans*, the Pearson correlation coefficient was calculated. Ten compounds with the highest correlation with lactic acid content were selected from all those analysed (Figure 3). The negative correlation compounds were ethyl hexanoate, ethyl octanoate, ethyl butanoate, 2-methylbutanol and ethyl heptanoate while the positive correlation compounds were acetaldehyde, isobutanol, 2-phenylethanol, acetoin and dodecanol. Esters, which are responsible for the fruity aroma of wine, were the main compounds with a negative correlation; as the concentration of lactic acid increased, the concentration of these compounds decreased. As discussed previously, this may be due to lower fatty acid production, since most of these esters are fatty acid ethyl esters, as reported by Vicente et al. [36], or to lower esterase activity, which is a possibility [45]. In contrast, the compounds with the highest positive correlations are mainly higher alcohols. These compounds are synthesised to a greater extent by *L. thermotolerans*. Their synthesis is related to readily assimilable nitrogen. Using a YAN-poor medium or yeast with higher nitrogen requirements results in more higher alcohols being synthesised from the corresponding keto acids [12]. Acetaldehyde and acetoin are associated with glycerol–pyruvate fermentation, which restores the altered redox potential resulting from lactic acid production [10].

Several studies indicate that the required value of readily assimilable nitrogen for *L. thermotolerans* should be greater than 200 mg/L [46]. Additionally, lactic acid production is closely related to the amount of available YAN in the must, reaching its maximum at 180–200 mg/L and above. Production is much lower in more stressful environments (below 100 mg/L) [47], similar to the requirements of *S. cerevisiae* for normal fermentation. Below 100 mg/l of YAN, sluggish fermentations and even stuck fermentations can occur. Since the amount of available nitrogen remained constant throughout, the results imply that *L. thermotolerans* possesses a greater ability to synthesise these compounds and/or has higher YAN requirements. This makes the medium relatively poorer, as the yeast’s needs are greater. In this sense, some authors have observed higher production of butanol, 2-methylbutanol and 3-methylbutanol in many *L. thermotolerans* strains than in an *S. cerevisiae* control strain [36]. This is partially consistent with the findings of this study, since 2-methylbutanol shows a negative correlation, which may be due to a lower amount of its amino acid precursor, isoleucine, in the must [12].

### 3.5. Organoleptic Characterization

A sensory analysis of all the wines was carried out, in which the tasting panel was asked to rate the aroma intensity and taste balance on a hedonic scale from 1 to 10. They were also asked to rate the acidity on a scale of −5 to 5, where −5 is very slightly acidic, 5 is excessively acidic and 0 is the optimum value. Finally, the tasters had to give an overall score to the wine, considering all of its organoleptic attributes, on a hedonic scale of zero to ten.

**Figure 4:**
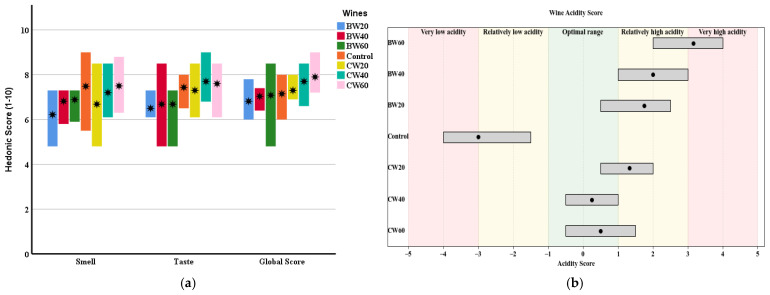
(**a**) Max–Min chart of the tasting scores for the wines. The central dot represents the median va. (**b**) Max–Min chart of the acidity tasting scores for the wines. The central dot represents the median value. Control: control wine; BW20: 20 h base wine, inoculation of *S. cerevisiae* 20 h after *L. thermotolerans*; BW40: 40 h base wine, inoculation of *S. cerevisiae* 40 h after *L. thermotolerans*; BW60: 60 h base wine, inoculation of *S. cerevisiae* 60 h after *L. thermotoleran*s CW20: coupage wine made with BW20 and control wine (80–20%); CW40: coupage wine made with BW40 and control wine (55–45%); CW60: coupage wine made with BW60 and control wine (50–50%).

Figure 4 shows the median, maximum and minimum scores given to the wines in terms of smell, taste and overall quality. Base wines made with *L. thermotolerans* are rated worse than the control wine in terms of smell and taste. This is directly related to the results of the volatile compound and aromatic series analysis. *L. thermotolerans* produces fewer esters in these wines, resulting in a lower OAV in the fruity and green fruit series. These series are associated with positive aromas in wine. However, the coupage wines show a clear improvement, particularly CW40 and CW60. Similar to the base wines, the coupage partially restores the ester content of the base wines, resulting in higher OAV values in the fruity and green fruit series. Also, higher acidity improves the overall assessment of these wines. Without being excessive, better acidity causes the tasters to score these wines higher than the control wine in the global score. Tasters rated these wines as livelier and more expressive on the palate thanks to the enhanced acidity.

## 4. Conclusions

The use of *Lachancea thermotolerans* has been confirmed as an effective way to naturally acidify wine through the production of lactic acid and reducing its pH. However, this acidification adversely affects the production of volatile compounds, particularly the esters responsible for fruity and floral aromas, such as isoamyl acetate, ethyl butanoate, ethyl hexanoate and 2-phenylethyl acetate. These compounds decreased as lactic acid increased. Conversely, the levels of compounds such as acetoin, acetaldehyde, dodecanol and isobutanol increased, potentially resulting in a loss of aromatic freshness. Nevertheless, blending wines fermented with *L. thermotolerans* with control wines mitigated these effects, as the improvement in acidity was retained while the lost aromatic complexity was recovered. This was particularly evident when base wines made with *L. thermotolerans* inoculated 40–60 h before *S. cerevisiae* were used. These blends offered a more balanced sensory profile with increased fruity, floral, citrus and creamy notes compared to base wines. They were also better rated from a sensory point of view. Overall, the strategic combination of sequential fermentations and coupages improves the wine’s freshness and aromatic intensity. This makes *L. thermotolerans* a valuable asset in winegrowing regions affected by global warming and a subsequent loss of natural acidity.

## Figures and Tables

**Table 1:** Oenological variables and color parameters of the base wines.

	Control	BW20	BW40	BW60
pH	4.01 ± 0.02 a	3.62 ± 0.02 b	3.6 ± 0.02 b	3.62 ± 0.01 b
Titratable Acidity (g/L TH2)	3.65 ± 0.05 d	6.01 ± 0.06 c	7.06 ± 0.04 b	7.26 ± 0.02 a
Ethanol (%*v*/*v*)	13.8 ± 0.1 a	13.33 ± 0.06 b	13.07 ± 0.06 c	12.8 ± 0.1 d
Volatile Acidity (g/L AcH)	0.44 ± 0.02 c	0.46 ± 0.05 bc	0.59 ± 0.07 ab	0.7 ± 0.07 a
Lactic Acid (g/L)	0.7 ± 0.2 c	2.5 ± 0.2 b	3.47 ± 0.08 a	3.8 ± 0.08 a
Malic Acid (g/L)	0.27 ± 0.02 c	0.29 ± 0.02 b	0.54 ± 0.03 a	0.52 ± 0.05 a
Glycerin (g/L)	4.7 ± 0.3 d	5.4 ± 0.1 c	6.9 ± 0.2 a	6.1 ± 0.2 b
Color Index	10.9 ± 0.4 b	12.6 ± 0.03 a	12.88 ± 0.06 a	13.05 ± 0.05 a
Tonality	36.58 ± 0.07 d	39.6 ± 0.4 c	45.7 ± 0.3 b	47.06 ± 0.09 a
Total Polyphenol Index	23.77 ± 0.06 c	25.2 ± 0.8 b	26 ± 0.1 ab	27 ± 0.3 a

TH_2_: Tartaric acid; AcH: acetic acid; BW20: *L. thermotolerans* followed by inoculation of *S. cerevisiae* after 20 h; BW40: *L. thermotolerans* followed by inoculation of *S. cerevisiae* after 40 h; BW60: *L. thermotolerans* followed by inoculation of *S. cerevisiae* after 60 h. Different letters indicate significant differences at 95% confidence level.

**Table 2:** Volatile aroma compounds determined in the base wines.

	Control	BW20	BW40	BW60
**Alcohol**				
**Major Alcohols (mg/L)**	**518 ± 7 c**	**558 ± 4 b**	**609 ± 10 a**	**514 ± 16 c**
Methanol	65 ± 5 a	55 ± 3 b	57 ± 1 ab	61 ± 5 b
Propanol	62 ± 2 c	73 ± 1 a	68 ± 1 b	56 ± 1
Isobutanol	51.6 ± 0.5 c	68 ± 1 b	85 ± 2 a	68 ± 2
2-methylbutanol	53.8 ± 0.1 a	45.6 ± 0.6 b	42.9 ± 0.6 c	33.9 ± 0.9 b
3-methylbutanol	266.7 ± 0.8 c	293 ± 2 b	326 ± 6 a	268 ± 6 b
2-phenylethanol	17.9 ± 0.3 c	24 ± 1 b	29.3 ± 0.9 a	26.1 ± 0.6 a
**Minor Alcohols (µg/L)**	**2896 ± 205**	**2742 ± 273**	**2792 ± 170**	**2414 ± 166**
Hexanol	2840 ± 204 a	2670 ± 271 ab	2712 ± 167 ab	2324 ± 163 b
2-ethyl-1-hexanol	39 ± 1 bc	50 ± 2 a	42 ± 2 b	38 ± 2 c
Octanol	7.78 ± 0 c	9.8 ± 0.03 b	13.7 ± 0.4 a	13.5 ± 0.2 a
Decanol	5 ± 0.4	N.D.	N.D.	N.D.
Dodecanol	2.7 ± 0.1 d	11.5 ± 0.3 c	22 ± 1 b	37 ± 3 a
Farnesol	1.9 ± 0.1 a	0.21 ± 0.02 b	1.7 ± 0.2 a	1.63 ± 0.01 a
**Esters**				
**Major Esters (mg/L)**	**101.9 ± 0.9 d**	**144 ± 1 c**	**186 ± 3 a**	**164 ± 3 a**
Ethyl acetate	61.3 ± 0.6 b	51 ± 1 c	69 ± 1 a	61 ± 1 c
Ethyl lactate	33.5 ± 0.1 d	86 ± 2 c	109 ± 2 a	95 ± 2 c
Diethyl succinate	7.2 ± 0.7 b	7.1 ± 0.3 b	8.4 ± 0.2 a	8.5 ± 0.3 c
**Minor Esters (µg/L)**	**4072 ± 118 a**	**1148 ± 23 b**	**964 ± 10 c**	**959 ± 25 c**
Ethyl isobutanoate	41 ± 1 c	37.7 ± 0.6 c	94 ± 7 a	65 ± 2 b
Ethyl butanoate	101 ± 5 a	45.3 ± 0.9 b	33.7 ± 0.9 c	29 ± 1 c
Butyl acetate	0.98 ± 0.08 b	1.3 ± 0.1 a	1.31 ± 0.07 a	0.95 ± 0.08 b
Ethyl 2-methylbutanoate	4.7 ± 0.4 a	2.4 ± 0.3 b	2.3 ± 0.1 b	2.47 ± 0.08 b
Ethyl 3-methylbutanoate	5.5 ± 0.2 a	1.9 ± 0.2 b	5.8 ± 0.1 a	2 ± 0.1 b
Isoamyl acetate	1248 ± 115 a	444 ± 18 b	308 ± 4 bc	257 ± 11 c
Ethyl hexanoate	595 ± 10 a	115.1 ± 0.4 b	7.6 ± 0.7 c	7.2 ± 0.2 c
Hexyl acetate	24.7 ± 0.5 a	2.9 ± 0.2 c	1.43 ± 0.07 d	4.1 ± 0.3 b
Ethyl heptanoate	2.11 ± 0.04 a	1.02 ± 0.02 b	0.24 ± 0.02 c	0.19 ± 0.02 c
Ethyl benzoate	0.27 ± 0.05 a	0.2 ± 0.01 a	0.13 ± 0.01 c	0.16 ± 0.01 b
Ethyl octanoate	558 ± 10 a	155 ± 4 b	76.2 ± 0.4 c	72.6 ± 0.9 c
Octyl acetate	2.7 ± 0.3 a	2.2 ± 0.1 ab	2.5 ± 0.2 a	1.8 ± 0.2 b
Ethyl phenylacetate	0.3 ± 0.03 c	0.21 ± 0.01 d	0.39 ± 0.01 b	0.6 ± 0.03 a
2-phenylethanol acetate	107 ± 9 a	86 ± 5 b	92 ± 4 ab	85 ± 6 b
Ethyl decanoate	711 ± 10 a	194 ± 3 d	236 ± 3 c	289 ± 3 b
Ethyl undecanoate	0.35 ± 0.02 c	0.45 ± 0.02 b	0.59 ± 0.02 a	0.57 ± 0.01 a
Ethyl tetradecanoate	13.5 ± 0.8 a	9 ± 0.7 b	6.8 ± 0.4 c	8.3 ± 0.8 bc
Phenethyl benzoate	0.84 ± 0.05	0.79 ± 0.06	0.8 ± 0.04	0.8 ± 0.03
Ethyl hexadecanoate	23 ± 1 a	21 ± 2 b	20.3 ± 0.8 b	25 ± 2 ab
**Aldehydes**				
**Major Aldehydes (mg/L)**	**61 ± 4 c**	**207 ± 2 b**	**224 ± 2 a**	**210 ± 5 b**
Acetaldehyde	61 ± 4 c	207 ± 2 b	224 ± 2 a	210 ± 5 b
**Minor Aldehydes (µg/L)**	**40 ± 0.7 a**	**27.2 ± 0.5 c**	**29 ± 0.5 b**	**24.5 ± 0.9 d**
Benzaldehyde	2 ± 0.2 a	2 ± 0.1 a	1.9 ± 0.2 a	0.95 ± 0.04 b
Hexanal	6.1 ± 0.3 a	5.4 ± 0.3 ab	5 ± 0.4 b	4.8 ± 0.3 b
Heptanal	0.61 ± 0.01 b	0.63 ± 0.01 b	0.83 ± 0.05 a	0.1 ± 0.01 c
Octanal	12.1 ± 0.2 a	1.23 ± 0.09 c	0.82 ± 0.03 c	1.14 ± 0.06 b
Nonanal	2.6 ± 0.2	2.6 ± 0.3	2.5 ± 0.1	2.1 ± 0.2
Decanal	4 ± 0.2 a	4.5 ± 0.5 a	4.7 ± 0.4 a	3 ± 0.05 b
Phenylacetaldehyde	11.6 ± 0.8 a	9.4 ± 0.7 b	12.4 ± 0.8 a	11.5 ± 0.6 a
4-methylbenzaldehyde	1.03 ± 0.09 b	1.4 ± 0.1 a	0.85 ± 0.06 c	0.82 ± 0.04 c
**Ketones**				
**Major Ketones (mg/L)**	**45 ± 4 c**	**97 ± 1 b**	**118.7 ± 0.8 a**	**114 ± 2 b**
Acetoin	45 ± 4 c	97 ± 1 b	118.7 ± 0.8 a	114 ± 2 ab
**Minor Ketones (µg/L)**	**0.67 ± 0.06 b**	**0.75 ± 0.06 b**	**1.96 ± 0.08 a**	**0.7 ± 0.03 c**
Benzophenone	0.44 ± 0.04 a	0.4 ± 0.04 a	0.38 ± 0.03 a	0.29 ± 0.02 b
3-Heptanone	0.23 ± 0.02 c	0.35 ± 0.02 b	1.58 ± 0.06 a	0.42 ± 0.01 b
**Volatile Phenols (µg/L)**	**14.1 ± 0.9 a**	**5.6 ± 0.3 b**	**1.41 ± 0.06 c**	**0.24 ± 0.03 d**
Guaiacol	14.1 ± 0.9 a	5.6 ± 0.3 b	1.41 ± 0.06 c	0.24 ± 0.03 d
**Furanic Compounds (µg/L)**	**25.8 ± 0.9 c**	**27.4 ± 0.4 a**	**21.4 ± 0.5 b**	**21.04 ± 0.02 b**
Pentylfuran	25.8 ± 0.9 c	27.4 ± 0.4 a	21.4 ± 0.5 b	21.04 ± 0.02 b
**Lactones (µg/L)**	**9.2 ± 0.8 b**	**11 ± 0.3 a**	**9.7 ± 0.2 c**	**8.1 ± 0.3 c**
γ-Nonalactone	7.2 ± 0.7 a	4.5 ± 0.2 b	2.9 ± 0.09 c	2.46 ± 0.06 c
γ-Decalactone	2 ± 0.2 c	6.6 ± 0.3 a	6.8 ± 0.2 a	5.7 ± 0.3 b
**Terpenes and Norisoprenoids (µg/L)**	**60 ± 2 a**	**48 ± 2 b**	**50 ± 2 b**	**48 ± 1 b**
Limonene	29 ± 2 a	19 ± 1 b	19.1 ± 0.9 b	17.9 ± 0.3 b
β-Citronellol	23 ± 1 a	17 ± 1 b	17 ± 1 b	15 ± 1 b
β-Damascenone	6.1 ± 0.2 d	10.7 ± 0.4 c	12.3 ± 0.2 b	13.5 ± 0.2 a
E-Geranyl acetone	0.94 ± 0.02 a	0.69 ± 0.01 a	0.48 ± 0.01 b	0.49 ± 0.03 b
Z-Geranyl acetone	1.65 ± 0.03 a	1.5 ± 0.03 b	1.48 ± 0.04 b	1.5 ± 0.05 b

BW20: *L. thermotolerans* followed by inoculation of *S. cerevisiae* after 20 h; BW40: *L. thermotolerans* followed by inoculation of *S. cerevisiae* after 40 h; BW60: *L. thermotolerans* followed by inoculation of *S. cerevisiae* after 60 h. Different letters indicate significant differences at 95% confidence level.

**Table 3:** Aromatic series values of control and base wines.

	Control	BW20	BW40	BW60
Fruity	177 ± 1 a	51.2 ± 0.7 b	31.4 ± 0.4 c	27.1 ± 0.4 d
Green fruit	49.9 ± 0.6 a	14 ± 0.1 b	6.28 ± 0.09 c	4.91 ± 0.03 d
Green	4.7 ± 0.2 ab	4 ± 0.1 c	4.9 ± 0.2 a	4.3 ± 0.2 b
Creamy	0.81 ± 0.05 c	1.53 ± 0.03 b	1.78 ± 0.01 a	1.62 ± 0.03 b
Citrus	11.9 ± 0.1 a	7.1 ± 0.6 b	7 ± 0.2 b	5.5 ± 0.1 c
Chemistry	25.1 ± 0.3 b	24.8 ± 0.1 b	28.8 ± 0.5 a	24.9 ± 0.5 b
Honey	3.3 ± 0.2 a	2.7 ± 0.2 b	3.5 ± 0.2 a	3.2 ± 0.2 a
Waxy	118 ± 2 a	35.6 ± 0.6 b	20.2 ± 0.3 c	18.4 ± 0.2 d
Floral	3.97 ± 0.06 c	4.9 ± 0.2 b	5.8 ± 0.1 a	5.5 ± 0.2 a

BW20: *L. thermotolerans* followed by inoculation of *S. cerevisiae* after 20 h; BW40: *L. thermotolerans* followed by inoculation of *S. cerevisiae* after 40 h; BW60: *L. thermotolerans* followed by inoculation of *S. cerevisiae* after 60 h. Different letters indicate significant differences at 95% confidence level.

**Table 4:** Oenological parameters of control and coupage wines.

	Control	CW20	CW40	CW60
pH	4.01 ± 0.02 a	3.68 ± 0.01 b	3.77 ± 0.01 b	3.82 ± 0.01 a
Titratable Acidity (g/L TH_2_)	3.65 ± 0.05 b	5.43 ± 0.07 a	5.48 ± 0.03 a	5.38 ± 0.07 a
Ethanol (%*v*/*v*)	13.8 ± 0.1 a	13.40 ± 0.06 b	13.45 ± 0.12 b	13.35 ± 0.06 b
Volatile Acidity (g/L AcH)	0.44 ± 0.02 c	0.49 ± 0.02 b	0.53 ± 0.03 a	0.58 ± 0.03 a
Lactic Acid (g/L)	0.7 ± 0.2 b	2.12 ± 0.06 a	2.24 ± 0.04 a	2.39 ± 0.04 a
Malic Acid (g/L)	0.27 ± 0.02 a	0.28 ± 0.02 a	0.27 ± 0.03 a	0.28 ± 0.02 a
Glycerin (g/L)	4.7 ± 0.3 c	5.1 ± 0.3 b	6.9 ± 0.5 a	5.6 ± 0.4 b
Color Index	10.9 ± 0.4 b	12.26 ± 0.03 a	12.5 ± 0.04 a	12.3 ± 0.2 a
Tonality	36.58 ± 0.07 b	39.2 ± 0.3 a	39.5 ± 0.6 a	39.8 ± 0.3 a
Total Polyphenol Index	23.77 ± 0.06 b	24.8 ± 0.1 b	26.2 ± 0.1 a	26.55 ± 0.07 a

TH_2_: tartaric acid; AcH: acetic acid; control: control wine; CW20: coupage wine made with BW20 and control wine (80–20%); CW40: coupage wine made with BW40 and control wine (55–45%); CW60: coupage wine made with BW60 and control wine (50–50%). Different letters indicate significant differences at 95% confidence level.

**Table 5:** Aromatic series values of control and coupage wines.

	Control	CW20	CW40	CW60
Fruity	177 ± 1 a	82 ± 1 c	83 ± 4 c	99 ± 4 b
Green fruit	49.9 ± 0.6 a	22 ± 1 b	25 ± 1 b	25 ± 1 b
Green	4.7 ± 0.2 c	5.8 ± 0.5 ab	5.3 ± 0.5 bc	6.6 ± 0.3 a
Creamy	0.81 ± 0.05 c	1.67 ± 0.05 b	1.88 ± 0.03 a	1.97 ± 0.03 a
Citrus	11.9 ± 0.2 a	7 ± 1 c	9.5 ± 0.7 b	10.1 ± 0.1 b
Chemistry	25.1 ± 0.3 b	24.1 ± 0.4 b	27 ± 0.4 a	26.2 ± 0.3 a
Honey	3.3 ± 0.2 b	3.8 ± 0.4 a	3.7 ± 0.2 a	4.4 ± 0.2 a
Waxy	118 ± 2 a	49 ± 3 c	46 ± 3 c	64 ± 2 b
Floral	3.97 ± 0.06 b	4.1 ± 0.1 b	4.9 ± 0.1 a	5.2 ± 0.2 a

Control: control wine; CW20: coupage wine made with BW20 and control wine (80–20%); CW40: coupage wine made with BW40 and control wine (55–45%); CW60: coupage wine made with BW60 and control wine (50–50%). Different letters indicate significant differences at 95% confidence level.

## Data Availability

The original contributions presented in this study are included in the article/Supplementary Materials. Further inquiries can be directed to the corresponding author.

## Supplementary Materials

The following supporting information can be downloaded at https://www.mdpi.com/article/10.3390/foods14162773/s1: Table S1. Major and minor volatile aroma compounds in coupage wines.; Table S2. Major and minor volatile aroma compounds identified in the wines.; Table S3. Odour descriptor, odour threshold (µg/L) and aroma series assigned to the volatile compounds identified in the analysed wines.

## Abbreviations

The following abbreviations are used in this manuscript:

GC-FID	Gas chromatography—flame ionization detector
GC-MS	Gas chromatography—mass spectrum detector
OAV	Odour activity value
PCA	Principal component analysis
TDS	Thermal desorption system
YAN	Yeast assimilable nitrogen
