# Supplementary material for: Innovative Fermentation Approach Employing Lachancea thermotolerans for the Selective Production of High-Acidity Wines, Designed for Blending with Low-Acidity Counterparts to Achieve Chemically and Organoleptically Balanced Final Compositions
Source: Foods. 2025 Aug 9;14(16):2773. doi: 10.3390/foods14162773 (PMC12385897; doi:10.3390/foods14162773)
Supplement: Supplementary file 1 [file foods-14-02773-s001.zip › foods-3741001-supplementary.pdf]

Table S1. Major and minor volatile aroma compounds in coupage wines.

	Control	CW20	CW40	CW60
**Alcohol**				
**Major Alcohols (mg/l)**	**518 ± 7 c**	**551 ± 3 b**	**590 ± 11 a**	**584 ± 10 a**
Methanol	65 ± 5 b	80,9 ± 0,8 a	73 ± 3 ab	81 ± 2 a
Propanol	62 ± 2 c	66,9 ± 0,5 a	66 ± 1 ab	63 ± 1 bc
Isobutanol	51,6 ± 0,5 d	59,5 ± 0,9 c	67 ± 2 a	63,3 ± 0,7 b
2-methylbutanol	53,8 ± 0,1 a	46,3 ± 0,9 c	49 ± 1 b	46,4 ± 0,6 c
3-methylbutanol	266,7 ± 0,8 b	278 ± 1 b	310 ± 5 a	306 ± 8 a
2-phenylethanol	17,9 ± 0,3 c	19,3 ± 0,8 b	25 ± 1 a	25,3 ± 0,7 a
**Minor Alcohols (μg/l)**	**2896 ± 205 a**	**2453 ± 65 b**	**2212 ± 235 b**	**2344 ± 114 b**
Hexanol	2840 ± 204 a	2379 ± 66 b	2138 ± 230 b	2260 ± 111 b
2-ethyl-1-hexanol	39 ± 1 b	44 ± 3 ab	46 ± 4 ab	50 ± 4 a
Octanol	7,78 ± 0,02 b	15 ± 2 a	13 ± 2 a	15,1 ± 0,7 a
Decanol	5 ± 0,4	N.D.	N.D.	N.D.
Dodecanol	2,7 ± 0,1 c	12,9 ± 0,2 b	13,2 ± 0,5 b	15 ± 0,4 a
Farnesol	1,9 ± 0,1 b	1,6 ± 0,1 b	2 ± 0,3 b	2,9 ± 0,2 a
**Esters**				
**Major Esters (mg/l)**	**101,9 ± 0,9 c**	**170 ± 1 b**	**181 ± 2 a**	**184,7 ± 0,6 a**
Ethyl acetate	61,3 ± 0,6 a	50,1 ± 0,1 b	62 ± 1 a	61 ± 1 a
Ethyl lactate	33,5 ± 0,1 c	112,1 ± 0,7 b	111 ± 1 b	117 ± 1 a
Diethyl succinate	7,2 ± 0,7	7,5 ± 0,6	8,2 ± 0,4	6,8 ± 0,7
**Minor Esters (μg/l)**	**4072 ± 118 a**	**1198 ± 37 c**	**1580 ± 24 b**	**1685 ± 30 b**
Ethyl isobutanoate	41 ± 1 b	125 ± 9 a	128 ± 5 a	119 ± 5 a
Ethyl butanoate	101 ± 5 a	64 ± 5 c	76 ± 4 b	78 ± 2 b
Butyl acetate	0,98 ± 0,08 d	1,91 ± 0,08 b	1,52 ± 0,08 c	3,3 ± 0,3 a
Ethyl 2-methylbutanoate	4,7 ± 0,4 b	6,7 ± 0,7 a	6 ± 0,5 ab	6,1 ± 0,5 a
Ethyl 3-methylbutanoate	5,5 ± 0,2 b	7,5 ± 0,5 a	7,2 ± 0,6 a	6,8 ± 0,4 a
Isoamyl acetate	1248 ± 115 a	381 ± 35 c	691 ± 38 b	679 ± 16 b
Ethyl hexanoate	595 ± 10 a	229 ± 14 c	280 ± 11 b	275 ± 22 b
Hexyl acetate	24,7 ± 0,5 a	1,6 ± 0,2 c	1,8 ± 0,2 c	4,3 ± 0,5 b
Ethyl heptanoate	2,11 ± 0,04 a	0,87 ± 0,01 b	0,44 ± 0,02 c	0,46 ± 0,02 c
Ethyl benzoate	0,27 ± 0,05 a	0,11 ± 0,01 b	0,11 ± 0,02 b	0,15 ± 0,02 b
Ethyl octanoate	558 ± 10 a	229 ± 12 c	205 ± 15 c	294 ± 14 b
Octyl acetate	2,7 ± 0,3 bc	1,9 ± 0,3 c	3 ± 0,6 b	5,5 ± 0,3 a
Ethyl phenylacetate	0,3 ± 0,03 d	0,64 ± 0,06 c	0,83 ± 0,08 b	1,09 ± 0,05 a
2-phenylethanol acetate	107 ± 9 a	60 ± 2 c	69 ± 7 bc	78 ± 2 b
Ethyl decanoate	711 ± 10 a	77,7 ± 0,4 d	111 ± 4 c	132 ± 5 b
Ethyl undecanoate	0,35 ± 0,02 c	0,39 ± 0,01 bc	0,45 ± 0,03 ab	0,47 ± 0,03 a
Ethyl tetradecanoate	13,5 ± 0,8 c	18 ± 1 ab	16,2 ± 0,4 b	19,3 ± 0,9 a
Phenethyl benzoate	0,84 ± 0,05 c	1,38 ± 0,03 b	1,57 ± 0,05 a	1,69 ± 0,08 a
Ethyl hexadecanoate	23 ± 1 c	52 ± 1 ab	51 ± 4 b	58 ± 3 a
**Aldehydes**				
**Major Aldehydes. (mg/l)**	**61 ± 4 b**	**158 ± 13 a**	**142 ± 4 a**	**75 ± 7 b**
Acetaldehyde	61 ± 4 b	158 ± 13 a	142 ± 4 a	75 ± 7 b
**Minor. Aldehydes (μg/l)**	**40 ± 0,7 ab**	**35 ± 4 b**	**36 ± 2 b**	**43 ± 2 a**
Benzaldehyde	2 ± 0,2 ab	2,3 ± 0,2 ab	1,7 ± 0,4 b	2,7 ± 0,3 a

	Control	CW20	CW40	CW60
Hexanal	6,1 ± 0,3 b	5,6 ± 0,3 b	6,5 ± 0,2 ab	7,3 ± 0,7 a
Heptanal	0,61 ± 0,01 b	2 ± 0,2 a	0,7 ± 0,8 b	1,9 ± 0,1 a
Octanal	12,1 ± 0,2 a	2,1 ± 0,2 d	2,7 ± 0,3 c	4,2 ± 0,3 b
Nonanal	2,6 ± 0,2 b	4 ± 0,3 a	4,6 ± 0,6 a	4,9 ± 0,6 a
Decanal	4 ± 0,2 b	3,7 ± 0,4 b	5,4 ± 0,6 a	5,3 ± 0,5 a
Phenylacetaldehyde	11,6 ± 0,8 b	14 ± 2 ab	13,7 ± 0,9 ab	16,2 ± 0,8 a
4-methylbenzaldehyde	1,03 ± 0,09 ab	1,4 ± 0,2 a	1,1 ± 0,2 a	0,7 ± 0,1 b
**Ketones**				
**Major Ketones (mg/l)**	**45 ± 4 c**	**89 ± 2 b**	**117 ± 2 a**	**105 ± 5 a**
Acetoin	45 ± 4 c	89 ± 2 b	117 ± 2 a	105 ± 5 a
**Cetonas Minor. (μg/l)**	**0,67 ± 0,06 c**	**1,27 ± 0,09 b**	**1,16 ± 0,02 b**	**1,8 ± 0,2 a**
Benzophenone	0,44 ± 0,04 a	0,21 ± 0,01 b	0,22 ± 0,03 b	0,29 ± 0,02 b
3-Heptanone	0,23 ± 0,02 c	1,1 ± 0,1 b	0,93 ± 0,03 b	1,5 ± 0,2 a
**Volatile Phenols (μg/l)**	**14,1 ± 0,9 a**	**3,3 ± 0,6 c**	**2 ± 0,2 c**	**4,8 ± 0,3 b**
Guaiacol	14,1 ± 0,9 a	3,3 ± 0,6 c	2 ± 0,2 c	4,8 ± 0,3 b
**Furanic Compounds (μg/l)**	**25,8 ± 0,9 a**	**12,4 ± 0,9 b**	**12 ± 2 b**	**14 ± 1 b**
Pentylfuran	25,8 ± 0,9 a	12,4 ± 0,9 b	12 ± 2 b	14 ± 1 b
**Lactones (μg/l)**	**9,2 ± 0,8 c**	**12 ± 1 bc**	**13 ± 2 b**	**17,3 ± 0,3 a**
G-Nonalactone	7,2 ± 0,7 ab	5 ± 1 c	5,5 ± 0,6 bc	8,4 ± 0,7 ab
G-Decalactone	2 ± 0,2 c	6,97 ± 0,09 b	7 ± 1 b	8,8 ± 0,6 a
**Terpenes & Norisoprenoids (μg/l)**	**60 ± 2 a**	**50 ± 5 b**	**52 ± 2 ab**	**53 ± 1 ab**
Limonene	29 ± 2 a	19 ± 2 b	22,6 ± 0,6 b	22 ± 1 b
B-Citronellol	23 ± 1	19 ± 3	18 ± 1	18,2 ± 0,9
B-Damascenone	6,1 ± 0,2 c	8,1 ± 0,2 b	8,7 ± 0,4 ab	9,5 ± 0,6 a
E-Geranyl acetone	0,94 ± 0,02 b	2 ± 0,3 a	0,7 ± 0,1 b	1 ± 0,1 b
Z-Geranyl acetone	1,65 ± 0,03 b	2 ± 0,1 a	2 ± 0,2 ab	1,97 ± 0,09 ab

Table S2. Major and minor volatile aroma compounds identified in the wines.

Compound	Prv‡	CAS[c]	LRI[a]	LRI[b]	Slope	Interception	R^2	LOD[d] (µg/L)	LOQ[e] (µg/L)
**Alcohols**									
**Mayor Alcohols**									
Methanol*	M	67-56-1	925	879	5.04E-03	6.0E-03	0.995	1,1E+04	3,8E+04
Propanol*	S	71-23-8	1068	1060	9.1E-03	5.0E-03	0.995	1,3E+04	4,4E+04
Isobutanol*	S	78-83-1	1126	1108	9.5E-03	3.0E-03	0.999	1,4E+04	4,7E+04
Isoamyl alcohols†*	M	123-51-3	1243	1230	8.7E+03	9.0E-03	0.999	1,0E+05	3,3E+05
2-phenylethanol*	S	60-12-8	2011	1892	1,10E-02	0,002	0.999	1,3E+04	4,2E+04
**Minor Alcohols**									
Hexanol	F	111-27-3	867	867	1.00E-03	0.00E+00	0.944	1,1E+03	3,6E+03
2-ethyl-1-hexanol	S	104-76-7	1028	1027	1.04E-03	8.30E-06	0.955	8,7E+00	2,9E+01
Octanol	M	111-87-5	1060	1064	8,51E-05	7,24E-03	0,949	6,2E+00	2,1E+01
Decanol	M	123-51-3	1243	1230	8.7E+03	9.0E-03	0.999	2,6E+00	8,7E+00
Dodecanol	S	112-53-8	1461	1469	2.60E-03	8.65E-02	0.982	1,6E-01	5,2E-01
Farnesol	M	4602-84-0	1728	1740	6.65E-003	2.97E-01	0.992	2,1E+00	6,9E+00
**Esters**									
**Mayor Esters**									
Ethyl acetate*	S	141-78-6	906	885	4.96E-03	-3.0E-04	0.998	1,5E+04	5,1E+04
Ethyl lactate*	S	97-64-3	1354	1326	5.7E-03	0,03	0.999	3,6E+04	1,2E+05
Diethyl succinate*	S	123-25-1	1730	1702	5.8E-03	2.8E-02	0.996	4,7E+03	1,6E+04
**Minor Esters**									
Ethyl isobutanoate	S	97-62-1	753	755	8.22E-02	1.64E-02	0.989	2,9E+01	9,6E+01
Ethyl butanoate	S	105-54-4	802	802	7.80E-03	1.03E-03	0.989	1,2E+01	4,1E+01
Butyl acetate	S	123-86-4	823	819	7,06E-03	3,34E-02	0,978	9,0E-01	3,0E+00
Ethyl 2-methylbutanoate	M	7452-79-1	847.4	846	1,66E-03	4,19E-03	0,987	1,2E+00	4,0E+00

Compound	Prv‡	CAS[c]	LRI[a]	LRI[b]	Slope	Interception	R^2	LOD[d] (μg/L)	LOQ[e] (μg/L)
Ethyl 3-methylbutanoate	S	108-64-5	851	847	7.78E-03	1.03E-02	0.989	2,0E+00	6,6E+00
Isoamyl acetate	F	123-92-2	875	876	4.80E-02	1.12E-02	0.984	1,4E+02	4,5E+02
Ethyl hexanoate	F	123-66-0	1000	1001	7.02E-02	4.32E-02	0.917	2,6E+01	8,7E+01
Hexyl acetate	S	142-92-7	1014	1015	1.34E-01	1.65E-02	0.991	3,6E+00	1,2E+01
Ethyl heptanoate	M	106-30-9	1102	1095	3,69E-02	5,71E-03	0,991	1,3E-01	4,3E-01
Ethyl benzoate	M	93-89-0	1170	1176	7.10E-03	8.79E-3	0.979	2,97 E-02	9,9 E-02
Ethyl octanoate	F	106-32-1	1198	1196	1.78E-01	-4.70E-03	0.988	1,4E+01	4,5E+01
Octyl acetate	M	112-14-1			1.21E-02	3.91E-03	0.949	4,9 E-01	1,6 E+00
Ethyl phenylacetate	M	101-97-3	1244.95	1252	4,51E-03	1,40E-02	0,975	4,4E-01	1,5E+00
2-phenylethyl acetate	S	103-45-7	1256	1256	6.22E-02	4.10E-03	0.995	9,4E+01	3,1E+02
Ethyl decanoate	F	110-38-3	1395	1397	1.69E-01	6.90E-03	0.976	1,1E+01	3,6E+01
Ethyl undecanoate	M	627-90-7	1498	1495	1.14E-02	-8.24E-03	0.988	9,5 E-02	3,2 E-01
Phenethyl hexanoate	M	6290-37-5	1654	1646	5,31E-02	-2,34E-02	0,992	6,2E-02	2,1E-01
Ethyl tetradecanoate	F	124-06-1	1793	1793	1.84E-01	6.51E-03	0.987	1,7E+00	5,6E+00
Phenethyl benzoate	S	94-47-3	1859	1859	4,06E-02	-3,15E-02	0,985	2,9E-01	9,8E-01
Ethyl hexadecanoate	F	628-97-7	1992	1996	1.83E-01	-2.60E-03	0.971	5,8E+00	1,9E+01
**Aldehydes**									
**Mayor Aldehydes**									
Acetaldehyde*	S	75-07-0	688	800	4.23E-03	-7.0E-04	0.999	1,7E+04	5,5E+04
**Minor Aldehydes**									
Benzaldehyde	S	100-52-7	958.9	959	5.10E-03	7.00E-03	0.988	3,2E-01	1,1E+00
Hexanal	M	66-25-1	808	800	3,08E-03	3,76E-02	0,926	1,1E+00	3,8E+00
Heptanal	S	111-71-7	903	901	7.90E-03	2.56E-03	0.961	1,2E-01	3,9E-01
Octanal	S	124-13-0	1004	1004	9.10E-03	1.80E-02	0.961	3,2E-01	1,1E+00
Nonanal	S	124-19-6	1104	1103	9.90E-03	1.79E-02	0.979	1,4E-01	4,5E-01
Decanal	S	112-31-2	1206	1206	8.90E-03	1.78E-02	0.959	2,9E-01	9,5E-01

Compound	Prv‡	CAS[c]	LRI[a]	LRI[b]	Slope	Interception	R^2	LOD[d] (µg/L)	LOQ[e] (µg/L)
Phenylacetaldehyde	M	122-78-1	1046	1050	6,25E-04	3,56E-03	0,965	5,3E+00	1,8E+01
4-Methylbenzaldehyde	M	104-87-0			6.70E-04	9.59E-03	0.959	1,89 E-01	6,3 E-01
**Ketones**									
**Mayor ketones**									
Acetoin*	S	513-86-0	1337	1309	5.08E-03	-0,001	0.997	1,7E+04	5,7E+04
**Minor ketones**									
Benzophenone	M	119-61-9	1626	1625	1,27E-02	9,19E-02	0,993	1,5E-01	5,0E-01
3-Heptanone	S	106-35-4	1130	1141	3.28E-003	2.88E-02	0.910	3,8E-02	1,3E-01
**Volatile Phenols**									
Guaiacol	S	90-05-1	1091	1088	3.30E-03	1.50E-03	0.993	6,2E-02	2,1E-01
**Furanic Compounds**									
Pentilfuran	M	3777-69-3			7.53E-04	5,79E-02	0.981	2,97 E+00	9,9 E+00
**Lactones**									
γ-Nonalactone	M	104-61-0	1363	1362	4.90E-03	-1.00E-04	0.943	6,6E+00	2,2E+01
γ-Decalactone	S	706-14-9	1470	1470	7.34E-02	-1.31E-02	0.983	5,0E+00	1,7E+01
**Terpenes & Norisoprenoids**									
Limonene	S	138-86-3	1022	1030	3.30E-02	2.70E-03	0.976	8,5E+00	2,8E+01
β-Citronellol	M	106-22-9	1174	1169	1.99E-04	-3.50E-3	0.959	13,5 E+00	4,05 E+00
β-Damascenone	S	23726-93-4	1385	1388	1.13E-02	5.85E-03	0.976	1,1E+00	3,7E+00
E-Geranyl acetone	S	689-67-8	1442	1450	5.64E-03	6.32E-03	0.976	1,6E-01	5,5E-01
Z-Geranyl acetone	S	689-67-8	1442	1450	5.64E-03	6.32E-03	0.976	5,3E-01	1,8E+00

LRI: Linear retention index using definition of Van den Dool and Kratz (1963) in a HP-5MS capillary column (30 m/0.25 mm/0.25 µm. He) and *CPWAX57-CB capillary column (60 m/0.25 mm/0.40 µm. He). . aCalculated values bData collected from the NIST Webbook of Chemistry. http://webbook.nist.gov/chemistry. cCAS: Chemical Abstracts Service number. d LOD: Limit of Detection. e LOQ: Limit of Quantification. † Isoamyl alcohols = 2-methylbutanol + 3-methylbutanol.‡Prv: Standard providers. F: Fluka; M: Merck; S: Sigma-Aldrich.

Table S3. Odor descriptor, odor threshold (μg/L) and aroma series assigned to the volatile compounds identified in the analysed wines.

Compound	OPT	Descriptors	Serie	Reference
**Alcohols**				
**Mayor Alcohols**				
Methanol	668000	Chemical, medicinal, fruity	5	(Ogawa et al., 2022)
Propanol	830000	Ripe fruit, fusel alcohol	5	(Ogawa et al., 2022)
Isobutanol	40000	Nail polish, bitter	5	(Ogawa et al., 2022)
Isoamyl alcohols	30000	Burnt, alcohol	5	(Ogawa et al., 2022)
2-phenylethanol	10000	Rose, honey, lilac	9	(Ogawa et al., 2022)
**Minor Alcohols**				
Hexanol	8000	Green, grass, oily	3	(López de Lerma et al., 2018)
2-ethyl-1-hexanol	8000	Citrus, fresh	5	(Zhang et al., 2019)
Octanol	800	Waxy, green, citrus, aldehydic and floral	8	(Peinado et al., 2006)
Decanol	400	aldehydic waxy green	8	(Qu et al., 2024)
Dodecanol	1000	waxy, earthy, soapy, aldehydic	8	(Li et al., 2008)
Farnesol	20	floral juicy	9	(Muñoz-Castells et al., 2024)
**Esters**				
**Mayor Esters**				
Ethyl acetate	7500	Fruity, glue	5	(Guth, 1997)
Ethyl lactate	150000	Fruity, byttery	4	(Peinado et al., 2004)
Diethyl succinate	100000	Overripe melon	1	(Ogawa et al., 2022)
**Minor Esters**				
Ethyl isobutanoate	15	Sweet, etherial and fruity	1	(López de Lerma et al., 2018)
Ethyl butanoate	20	Fruity, floral	1	(Guth, 1997)
Butyl acetate	66	Sweet, ripe, banana, ethereal	1,6	(Takeoka et al., 1996)
Ethyl 2-methylbutanoate	18	sharp sweet green apple fruity	1,2	(Ferreira et al., 2000)

Compound	OPT	Descriptors	Serie	Reference
Ethyl 3-methylbutanoate	3	Pinneaple	1,2	(Ferreira et al., 2000)
Isoamyl acetate	30	sweet fruity banana	1	(Guth, 1997)
Ethyl hexanoate	14	sweet fruity pineapple green banana	1,2	(Ferreira et al., 2000)
Hexyl acetate	70	fruity green apple banana sweet	1,2	(SWIEGERS et al., 2005)
Ethyl heptanoate	2,2	fruity pineapple	1	(López de Lerma et al., 2018)
Ethyl benzoate	60	Minty, Fruity	1	(Buttery et al., 1988)
Ethyl octanoate	5	Banana, pineapple	1,8	(López de Lerma et al., 2018)
Ethyl phenylacetate	250	Rose, floral	7,9	*
Octyl acetate	20	Floral	9	(Cometto-Muñiz et al., 2008)
2-Phenylethyl acetate	250	floral-rose and gardenia	7,9	(Guth, 1997)
Ethyl decanoate	200	Sweet, fruity, caramel, grape	1,8	(Gómez-Míguez et al., 2007)
Ethyl undecanoate	200	Waxy, soapy	8	***
Phenethyl hexanoate	250	Fruity-green, fresh pineapple-like	8,9	*
Ethyl tetradecanoate	4000	Tropical fruit	8	(Moreno, 2005)
Phenethyl benzoate	250	Rose, honey, balsamic	2,9	*
Ethyl hexadecanoate	2000	Waxy, fruity nuances	8	(Moreno, 2005)
**Aldehydes**				
**Mayor Aldehydes**				
Acetaldehyde	110000	Pungent, ripe apple	1,5	(Moreno, 2005)
**Minor Aldehydes**				
Benzaldehyde	350	Bitter almond, smoked	1	(Buttery et al., 1988)
Hexanal	5	Herbaceous, green apple	3	(Buttery et al., 1988)
Heptanal	3	Herbal, coriander	3	(Buttery et al., 1988)
Octanal	2,5	Citrus, green, fresh, waxy	5	(López de Lerma et al., 2018)
Nonanal	2,5	Citrus	5	(López de Lerma et al., 2018)
Decanal	1,25	Citrus	5,8	(López de Lerma et al., 2018)

Compound	OPT	Descriptors	Serie	Reference
Phenylacetaldehyde	4	green sweet honey	3,7	(Buttery et al., 1971)
4-Methylbenzaldehyde	350	Fruity, Cherry, phenolic	1	**
**Ketones**				
**Mayor ketones**				
Acetoin	150000	Yogurt, butterscotch	4	(Zhang et al., 2015)
**Minor ketones**				
Benzophenone	65	Balsamic, rose, herbal, geraniun	9	
3-Heptanone	7,5	Green, ketonic	3	(Burdock and Fenaroli, 2010)
**Volatile Phenols**				
Guaiacol	75	Smoke, chemistry	6	(Rocha et al., 2004)
**Furanic Compounds**				
Pentylfuran	6	Fruity, green, vegetable	2	(Leffingwell & Associates, n.d.)
**Lactones**				
γ -Nonalactone	30	Coconut, creamy	1,4	(Ferreira et al., 2000)
γ -Decalactone	77.7	Peach, milky	1,4	(Gottmann et al., 2023)
**Terpenes & Norisoprenoids**				
Limonene	10	citrus orange fresh sweet	5,6	(López de Lerma et al., 2018)
β -Citronellol	40	Floral, Rose, citrus	9	(Leffingwell & Associates, n.d.)
β-Damascenone	4	natural rose	9	(Pineau et al., 2007)
E-Geranyl acetone	60	Magnolia, rose	9	(Zhu et al., 2019)
Z-Geranyl acetone	60	Magnolia, rose	9	(Zhu et al., 2019)

1 Fruity; 2 GreenFruit; 3 Green; 4 Creamy; 5 Citrus; 6 Chemistry; 7 Honey; 8 Waxy; 9 Floral. *Expressed as 2-phenethyl acetate; ** Expressed as Benzaldehyde; *** Expressed as Ethyl decanoate.

References

1. Burdock, G.A.; Fenaroli, G. *Fenaroli's Handbook Of Flavor Ingredients*, 6th ed; CRC Press: Boca Raton, FL, USA, 2010; ISBN 9780429150838.
2. Buttery, R.G.; Seifert, R.M.; Guadagni, D.G.; Ling, L.C. Characterization of additional volatile components of tomato. *J. Agri. Food Chem*. **1971**, *19*, 524–529. https://doi.org/10.1021/jf60175a011
3. Buttery, R.G.; Turnbaugh, J.G.; Ling, L.C. Contribution of volatiles to rice aroma. *J. Agri. Food Chem*. **1988**, *36*, 1006–1009. https://doi.org/10.1021/jf00083a025
4. Cometto-Muñiz, J.E.; Cain, W.S.; Abraham, M.H.; Gil-Lostes, J. Concentration-detection functions for the odor of homologous n-acetate esters. *Physiol. Behav*. **2008**, *95*, 658–667. https://doi.org/10.1016/J.PHYSBEH.2008.09.021
5. Ferreira, V.; López, R.; Cacho, J.F. Quantitative determination of the odorants of young red wines from different grape varieties. *J. Sci. Food Agric*. **2000**, *80*, 1659–1667. https://doi.org/10.1002/1097-0010(20000901)80:11<1659::AID-JSFA693>3.0.CO;2-6
6. Gómez-Míguez, M.J.; Cacho, J.F.; Ferreira, V.; Vicario, I.M.; Heredia, F.J. Volatile components of Zalema white wines. *Food Chem*. **2007**, *100*, 1464–1473. https://doi.org/10.1016/j.foodchem.2005.11.045
7. Gottmann, J.; Vestner, J.; Fischer, U. Sensory relevance of seven aroma compounds involved in unintended but potentially fraudulent aromatization of wine due to aroma carryover. *Food Chem*. **2003**, *402*, 134160. https://doi.org/10.1016/j.foodchem.2022.134160
8. Guth, H. Quantitation and Sensory Studies of Character Impact Odorants of Different White Wine Varieties. *J. Agric. Food Chem*. **1997**, *45*, 3027–3032. https://doi.org/10.1021/jf970280a
9. Leffingwell & Associates. Odor Properties & Molecular Visualization. Available online: http://www.leffingwell.com/esters.htm (accessed on 2 January 2025).
10. Li, H.; Tao, Y.-S.; Wang, H.; Zhang, L. Impact odorants of Chardonnay dry white wine from Changli County (China). *Eur. Food Res. Technol*. **2008**, *227*, 287–292. https://doi.org/10.1007/s00217-007-0722-9
11. López de Lerma, N.; Peinado, R.A.; Puig-Pujol, A.; Mauricio, J.C.; Moreno, J.; García-Martínez, T. Influence of two yeast strains in free, bioimmobilized or immobilized with alginate forms on the aromatic profile of long aged sparkling wines. *Food Chem*. **2018**, *250*, 22–29. https://doi.org/10.1016/j.foodchem.2018.01.036
12. Moreno, J.A. Influencia del tipo de envejecimiento sobre el perfil aromático de vinos generosos andaluces. Ph.D. Thesis, University of Córdoba, Andalucía, Spain. 2005.
13. Muñoz-Castells, R.; Moreno, J.; García-Martínez, T.; Mauricio, J.C.; Moreno-García, J. Assessing the Impact of Commercial Lachancea thermotolerans Immobilized in Biocapsules on Wine Quality: Odor Active Compounds and Organoleptic Properties. *Fermentation* **2024**, *10*, 303. https://doi.org/10.3390/fermentation10060303
14. Ogawa, M.; Vararu, F.; Moreno-Garcia, J.; Mauricio, J.C.; Moreno, J.; Garcia-Martinez, T. Analyzing the minor volatilome of Torulaspora delbrueckii in an alcoholic fermentation. *Eur. Food Res. Technol*. **2022**, *248*, 613–624. https://doi.org/10.1007/s00217-021-03910-y
15. Peinado, R.A.; Mauricio, J.C.; Moreno, J. Aromatic series in sherry wines with gluconic acid subjected to different biological aging conditions by Saccharomyces cerevisiae var. capensis. *Food Chem*. **2006**, *94*, 232–239. https://doi.org/10.1016/j.foodchem.2004.11.010

16. Peinado, R.A.; Moreno, J.; Bueno, J.E.; Moreno, J.A.; Mauricio, J.C. Comparative study of aromatic compounds in two young white wines subjected to pre-fermentative cryomaceration. *Food Chem*. **2004**, *84*, 585–590. https://doi.org/10.1016/S0308-8146(03)00282-6
17. Pineau, B.; Barbe, J.-C.; Van Leeuwen, C.; Dubourdieu, D. Which Impact for β-Damascenone on Red Wines Aroma? *J. Agri. Food Chem*. **2007**, *55*, 4103–4108. https://doi.org/10.1021/jf070120r
18. Qu, J.; Chen, X.; Wang, X.; He, S.; Tao, Y.; Jin, G. Esters and higher alcohols regulation to enhance wine fruity aroma based on oxidation-reduction potential. *LWT-Food Sci Technol* **2004**, *200*, 116165. https://doi.org/10.1016/j.lwt.2024.116165
19. Rocha, S.M.; Rodrigues, F.; Coutinho, P.; Delgadillo, I.; Coimbra, M.A. Volatile composition of Baga red wine. *Anal. Chim. Acta*. **2004**, *513*, 257–262. https://doi.org/10.1016/j.aca.2003.10.009
20. SWIEGERS, J.H.; BARTOWSKY, E.J.; HENSCHKE, P.A.; PRETORIUS, I.S. Yeast and bacterial modulation of wine aroma and flavour. *Aust. J. Grape Wine Res*. **2005**, *11*, 139–173. https://doi.org/10.1111/j.1755-0238.2005.tb00285.x
21. Takeoka, G.; Buttery, R.G.; Ling, L. Odour Thresholds of Various Branched and Straight Chain Acetates. *LWT-Food Sci Technol*. **1996**, *29*, 677–680. https://doi.org/10.1006/fstl.1996.0105
22. Zhang, S.; Petersen, M.; Liu, J.; Toldam-Andersen, T. Influence of Pre-Fermentation Treatments on Wine Volatile and Sensory Profile of the New Disease Tolerant Cultivar Solaris. *Molecules* **2015**, *20*, 21609–21625. https://doi.org/10.3390/molecules201219791
23. Zhang, Y.-S.; Du, G.; Gao, Y.-T.; Wang, L.-W.; Meng, D.; Li, B.-J.; Brennan, C.; Wang, M.-Y.; Zhao, H.; Wang, S.-Y.; Guan, W.-Q. The Effect of Carbonic Maceration during Winemaking on the Color, Aroma and Sensory Properties of 'Muscat Hamburg' Wine. *Molecules* **2019**, *24*, 3120. https://doi.org/10.3390/molecules24173120
24. Zhu, L.-X.; Zhang, M.-M.; Shi, Y.; Duan, C.-Q. Evolution of the aromatic profile of traditional Msalais wine during industrial production. *Int. J. Food Prop*. **2019**, *22*, 911–924. https://doi.org/10.1080/10942912.2019.1612428
